# A de novo genome assembly of *Solanum verrucosum* Schlechtendal, a Mexican diploid species geographically isolated from other diploid A-genome species of potato relatives

**DOI:** 10.1093/g3journal/jkac166

**Published:** 2022-07-01

**Authors:** Awie J Hosaka, Rena Sanetomo, Kazuyoshi Hosaka

**Affiliations:** Rhelixa Inc., Tokyo 101-0061, Japan; Kihara Institute for Biological Research, Yokohama City University, Yokohama 244-0813, Japan; Potato Germplasm Enhancement Laboratory, Obihiro University of Agriculture and Veterinary Medicine, Obihiro, Hokkaido 080-8555, Japan; Potato Germplasm Enhancement Laboratory, Obihiro University of Agriculture and Veterinary Medicine, Obihiro, Hokkaido 080-8555, Japan

**Keywords:** *Solanum verrucosum*, Mexican wild potato, genome assembly, A-genome species

## Abstract

There are over 100 known species of cultivated potatoes and their wild relatives. Many of these species, including cultivated potatoes, share the A genome; these species are mainly distributed in South America and are reproductively isolated from Mexican diploid species. The only diploid A-genome species distributed in Mexico is *Solanum verrucosum* Schlechtendal, which is also a maternal progenitor of Mexican polyploid species. In this study, we constructed a high-quality de novo assembly of the *S. verrucosum* genome using PacBio long-read sequencing and Hi-C scaffolding technologies. A monohaploid clone (2*n* = *x *=* *12) of *S. verrucosum* was used to reduce assembly difficulty due to the heterozygous nature of the species. The final sequence assembly consisted of 780.2 Mb of sequence, 684.0 Mb of which were anchored to the 12 chromosomes, with a scaffold N50 of 55.2 Mb. Putative centromeres were identified using publicly available data obtained via chromatin immunoprecipitation sequencing against a centromere-specific histone 3 protein. Transposable elements accounted for approximately 61.8% (482.1 Mb) of the genome, and 46,904 genes were functionally annotated. High gene synteny and similarity were revealed among the genomes of *S. verrucosum*, *Solanum commersonii*, *Solanum chacoense*, *Solanum phureja*, *Solanum tuberosum*, and *Solanum lycopersicum*. The reference-quality *S. verrucosum* genome will provide new insights into the evolution of Mexican polyploid species and contribute to potato breeding programs.

## Introduction

Potato (*Solanum tuberosum* L., 2*n *=* *4*x *=* *48) is the most important noncereal food crop in the world. High genetic diversity is observed among primitive cultivated potatoes and the over 100 wild potato species distributed from North and Central America to South America (Hawkes 1990; Spooner *et al.* 2014). These species are classified into 2 reproductively isolated groups: (1) a group including all Mexican diploid species except for *S. verrucosum* Schlechtendal and (2) a group including all Mexican polyploid species, *S. verrucosum*, and all South American species (Hawkes 1958). Based on the meiotic chromosome pairing of interspecific hybrids, the A genome is assigned to the species in the second group (Matsubayashi 1991). Since sexual hybrids between Mexican diploid species and A-genome species are extremely difficult to obtain, their genome affinity has long been debated (Matsubayashi 1991; Pendinen *et al.* 2008).

*S. verrucosum* is the only diploid A-genome species from Mexico and is assumed to contribute the A genome of Mexican polyploid species (Hosaka *et al.* 1984). Most diploid tuber-bearing *Solanum* species are self-incompatible (Pushkarnath 1942), whereas *S. verrucosum* is self-compatible (Hawkes 1990). *S. verrucosum* is cross-compatible with most South American species as the female parent (Eijlander *et al.* 2000) and with some Mexican diploid species, which provides an opportunity to transfer useful traits from Mexican diploid species to cultivated potatoes as a bridging species (Hermsen and Ramanna 1976; Jansky and Hamernik 2009). The Mexican species, including *S. verrucosum*, are valuable sources of disease and pest resistance in potato breeding (Hein *et al.* 2009; Chen *et al.* 2018).

The first potato genome was sequenced from the DM 1-3 516 R44 clone (hereafter referred to as DM) (Potato Genome Sequencing Consortium 2011). DM resulted from the chromosome doubling of a monoploid derived via anther culture of the cultivated diploid species *Solanum* *phureja* Juz. et Buk. (Lightbourn and Veilleux 2007). The homozygous nature of the clone facilitated genome sequencing. Since then, potato whole genomes have been sequenced mainly from cultivated potato species (Kyriakidou, Achakkagari, *et al.* 2020; Kyriakidou, Anglin, *et al.* 2020; van Lieshout *et al.* 2020; Zhou *et al.* 2020; Yan *et al.* 2021). Recent advances such as long-read sequencing coupled with high-throughput chromosome conformation capture (Hi-C) scaffolding technologies have resulted in great improvements in the DM genome (DM v6.1; Pham *et al.* 2020), and chromosome-scale phased assemblies have been obtained from heterozygous diploid and tetraploid potatoes (Zhou *et al.* 2020; Yan *et al.* 2021; Hoopes *et al.* 2022; Sun *et al.* 2022). However, whole-genome sequencing in wild species has been limited to *Solanum* *commersonii* Dun. (Aversano *et al.* 2015) and *Solanum* *chacoense* Bitt. (Leisner *et al.* 2018), both of which are distributed in the southern marginal distribution area of the South American A-genome species (Hawkes and Hjerting 1969). Only a draft genome sequence has been reported for the Mexican diploid species *S. pinnatisectum* Dunal (Tiwari *et al.* 2021).

In this study, we generate a chromosome-scale assembly of the genome of the Mexican diploid species *S. verrucosum* using PacBio long-read sequencing and Hi-C scaffolding technologies. A monohaploid *S. verrucosum* clone (2*n* = *x *=* *12) was used to reduce complexity caused by the heterozygous nature of the species. The constructed reference-quality genome will provide new insights into the evolutionary process in Mexican polyploid species and contribute to potato breeding programs.

## Materials and methods

### Plant material

A monohaploid clone of *S. verrucosum* (11H23, available as PI 666968 from the U.S. Potato Genebank) that was derived from anther culture (Irikura and Sakaguchi 1972) and maintained in vitro in our laboratory (Sanetomo and Hosaka 2021) was used for sequencing.

### DNA extraction

A plant grown in vitro was transferred to a pot filled with soil and further grown for DNA extraction. Fresh leaves were collected, frozen in liquid nitrogen, and ground into powder with a mortar and pestle. The powder was suspended in 7 ml of 2× CTAB buffer (100 mM Tris-Cl buffer pH 8.0, 20 mM EDTA pH 8.0, 1.4 M NaCl, 2% CTAB, 1% PVP-40, and 0.2% 2-mercaptoethanol) and incubated at 60°C for 30 min. The suspension was gently mixed with 5 ml of chloroform: isoamyl alcohol (24:1) and centrifuged at 10,000 rpm for 5 min at 20°C. Using a wide-bore pipet tip, the supernatant was transferred to a 50-ml tube containing 5 ml of isopropanol and mixed gently by inverting the tube. Aggregated DNA strands were hooked and drawn up using a Pasteur pipet modified by flaming the tip and bending it into a U shape, after which they were transferred to a tube containing 10 ml of 75% ethanol washed for 30 min. Then, the aggregated DNA was dissolved in 2 ml of TE buffer (10 mM Tris-Cl buffer pH 8.0 and 1 mM EDTA pH 8.0) and incubated with 5 μl of RNase (10 mg/ml) for 3 h at room temperature. After complete dissolution, 100 μl of 5 M NaCl and 700 μl of 99.9% ethanol were added, followed by mixing and incubation at 4°C overnight to precipitate polysaccharides. After centrifugation at 10,000 rpm for 5 min at 4 °C, the supernatant was collected and mixed gently with 9 ml of 75% ethanol with 10 mM ammonium acetate. The aggregated DNA strands were hooked and drawn up using a U-shaped Pasteur pipet, transferred to a tube containing 10 ml of 75% ethanol, and washed for 30 min. Then, the DNA was dried completely while hanging on the U-shaped Pasteur pipet and dissolved in 100 μl of sterile water.

### Genome sequencing and assembly

The quality of the extracted DNA was measured with a Genomic DNA ScreenTape System (Agilent) and a Qubit Fluorometer (Thermo Fisher Scientific). A long-read DNA library was prepared with the SMRTbell Express Template Prep Kit 2.0 (PacBio) and sequenced using the PacBio Sequel lle system in CCS mode (PacBio). The resulting raw data were converted to FASTQ format using BAM2fastx 1.3.1 (PacBio). Reads longer than 5 kb were extracted with SeqKit 0.15.0 (Shen *et al.* 2016) and used for genome assembly with the Hifiasm 0.15.5-r350 assembler (Cheng *et al.* 2021).The -l 0 option was specified to disable the purge haplotigs function since the plant was monohaploid.

### Hi-C sequencing and scaffolding

The Hi-C library was prepared using the Dovetail Omni-C Kit (Dovetail Genomics) following the Proximity Ligation Assay Nonmammalian Samples Protocol version 1.0. The prepared library was sequenced on the NovaSeq 6000 (Illumina) platform. The read quality was assessed using FastQC 0.11.8 (Andrews 2010) and MultiQC v1.8 (Ewels *et al.* 2016) and then filtered using Trimmomatic 0.39 (Bolger *et al.* 2014) with the “ILLUMINACLIP: TruSeq3-PE.fa : 2:30:10 TRAILING : 20 SLIDINGWINDOW : 4:15 HEADCROP : 10 MINLEN : 50” options. The trimmed reads were aligned to the contigs using Juicer 1.6 (Durand *et al.* 2016). Since DNase I was used to digest fixed nucleosomes, the “-s none -y none” options were specified. The generated contact maps were then used for scaffolding with a 3D-DNA pipeline (Dudchenko *et al.* 2017) with the default parameters. The scaffolds were manually corrected using JuiceBox 1.11.08 (https://github.com/aidenlab/Juicebox). The corrected scaffolds were aligned to the DM v6.1 reference genome using D-GENIES (Cabanettes and Klopp 2018). The identities and directions of the scaffolds were determined based on the alignment.

### Identification of organelle sequences

To identify regions or contigs derived from organelle genomes, sequences of chloroplast genome of *S. verrucosum* (MH021593.1; Huang *et al.* 2019) and mitochondrial genome of *S. tuberosum* cultivar Désirée (MN104801, MN104802, and MN104803; Varré *et al.* 2019) were obtained from the National Center for Biotechnology Information (NCBI), and a nucleotide homology search was performed against *S. verrucosum* contigs using BLASTN 2.12.0 (Altschul *et al.* 1990) with the “-outfmt 6 -evalue 0.0001” options. Regions with more than 10 kb of alignment length and with more than 90% homology were selected and reformatted to BED files. Overlapped regions were merged using BEDTools 2.30.0 (Quinlan and Hall 2010).

### Identification of putative centromeres

To identify centromeres, sequence reads generated via chromatin immunoprecipitation sequencing (ChIP-seq) against a centromere-specific histone 3 (CENH3) protein that were publicly available from NCBI were obtained for *S. verrucosum* (SRR18548893; Zhang *et al.* 2014) and *S. phureja* (SRR18548894; Gong *et al.* 2012) and aligned to their genomes using Bowtie2 (Langmead and Salzberg 2012) in single-end mode. The resulting BAM files were converted to BigWig files using DeepTools 3.5.1 (Ramírez *et al.* 2016) for visualization on the IGV 2.11.0 genome browser (Robinson *et al.* 2011). Centromeric regions of chromosomes were manually identified with IGV.

### Annotation

Transposable elements (TEs) were identified using EDTA 1.9.6 (Ou *et al.* 2019), and the defined TE regions were hard masked. To evaluate assembly completeness, the long terminal repeat (LTR) assembly index (LAI) score (Ou *et al.* 2018) was calculated using the EDTA output files. Tandem repeats were defined using Tandem Repeats Finder v4.09 (Benson 1999) with the default parameters, and the defined repeats were soft masked using BEDTools. The masked scaffolds were subjected to gene prediction using the MAKER 3.01.03 (Cantarel *et al.* 2008) annotation pipeline by providing mRNA and protein sequences of DM v6.1 (Pham *et al.* 2020) and pretrained AUGUSTUS (Stanke *et al.* 2004) gene models of tomato. The functional annotation of the predicted proteins was performed using Hayai-Annotation Plants v.2 (Ghelfi *et al.* 2019). The density of the annotated TE families, Miniature Inverted-repeat Transposable Element (MITE) derivatives, genes, and CENH3 ChIP-seq reads within every 1 Mb segment was calculated using BEDTools and visualized in a circular heatmap generated by Circos (Krzywinski *et al.* 2009).

### Genome synteny and orthologs

The genome of *S. verrucosum* was compared with those of *S. phureja* (DM v6.1; Pham *et al.* 2020), diploid *S. tuberosum* (Solyntus v1.1; van Lieshout *et al.* 2020), *S. chacoense* (M6; Leisner *et al.* 2018), *S. commersonii* (Aversano *et al.* 2015), and *Solanum* *lycopersicum* L. (Hosmani *et al.* 2019). Syntenic gene pairs were searched using MCScan (python version) (Tang *et al.* 2008) with the default parameters, and syntenic blocks containing more than 30 genes were visualized. The orthologous relationships of *S. verrucosum* genes were assessed using OrthoFinder (Emms and Kelly 2015, 2019). All protein-coding genes except for those encoding isoforms or sequences shorter than 10 amino acids were compared. Intersections of orthogroups were visualized with UpSetR 1.4.0 (Lex *et al.* 2014; Conway *et al.* 2017).

## Results and discussion

### Genome assembly

**Fig. 1.**
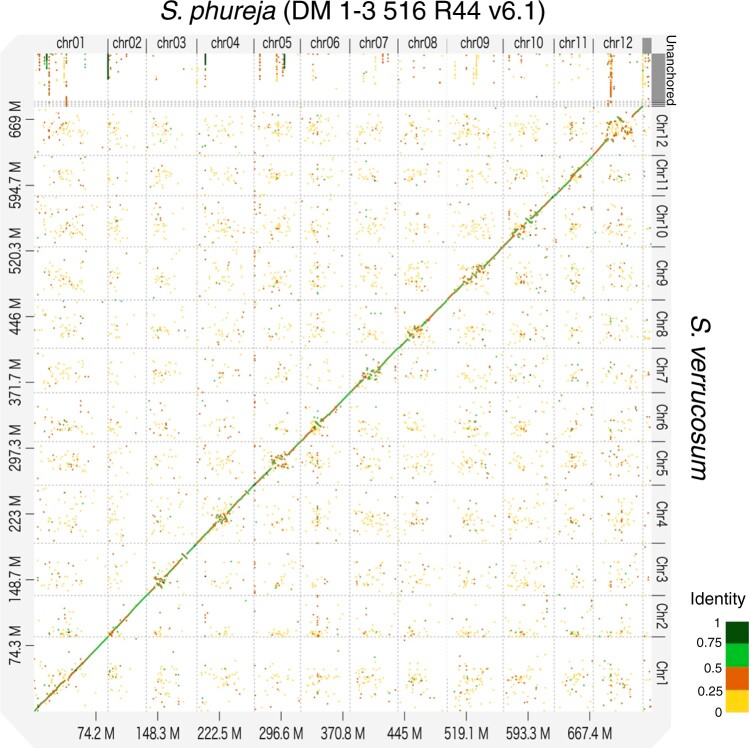
Dot plot analysis between *S. verrucosum* and *S. phureja* using D-GENIES with the “hide noise” option.

We obtained 46.5 Gb of HiFi reads using a PacBio Sequel IIe system with an N50 read size of 15.6 kb and an average read size of 14.9 kb. Reads longer than 5 kb were used for assembly with Hifiasm. The resulting assembly consisted of 1,437 contigs with an N50 contig size of 21.0 Mb (Table 1). The contigs were error corrected and scaffolded with 101 million Omni-C read pairs using Juicer and a 3D-DNA pipeline (Supplementary Fig. 1). The final sequence assembly consisted of 780.2 Mb, among which 684.0 Mb were anchored to the 12 chromosomes, with a scaffold N50 of 55.2 Mb (Table 1). Of the remaining 1,535 unanchored contigs (a total of 96.3 Mb in size), 688 contigs (33.3 Mb) and 102 contigs (3.6 Mb) showed high homology to the chloroplast and mitochondrial genomes, respectively (Supplementary File 1). The dot plot analysis using these contigs against the organelle genomes indicated that these contigs were fragments of the organelle genomes (Supplementary Fig. 2). This is in accordance with previous studies that most of smaller contigs from Hifiasm assembly corresponded to small portions of the chloroplast and mitochondrial genomes (Sharma *et al.* 2022; Sun *et al.* 2022). The other unanchored contigs (59.4 Mb) consisted mostly of TEs (88.6%) and showed homology to localized regions in the chromosomes (Supplementary File 1 and Fig. 1).

**Table 1.** Assembly statistics.

	Primary contigs with PacBio reads	Final scaffolded contigs after Hi-C sequencing
Number of contigs	1,437	1,547
Total size, bp	779,910,189	780,238,689
Longest size, bp	55,137,318	84,109,000
Mean size, bp	542,735	504,356
N50 size, bp	20,992,750	55,157,000

### Putative centromeres

**Fig. 2.**
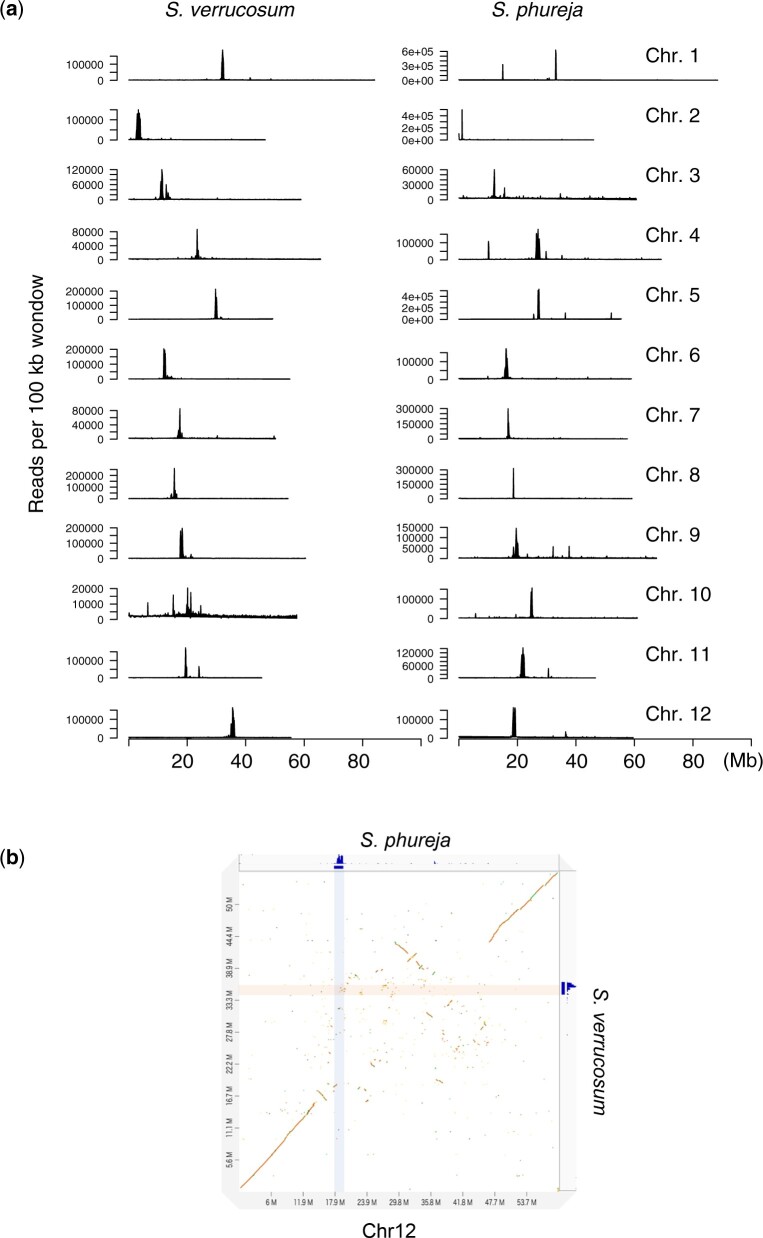
Putative centromeres. a) Distribution of CENH3 ChIP-seq signals in every 100 kb window in *S. verrucosum* and *S. phureja*. b) Dot plot between *S. verrucosum* and *S. phureja* for chromosome 12. ChIP-seq signals against the CENH3 proteins of *S. verrucosum* and *S. phureja* are shown on the right and at the top of the plot, respectively, and are highlighted on the plot.

The dot plot analysis performed between the genome of *S. verrucosum* and that of *S. phureja* DM v6.1 using D-GENIES showed significant consistency in the distal regions of each chromosome, while the central regions diverged considerably (Fig. 1). This is because centromere sequences evolve rapidly (Henikoff *et al.* 2001) and might be distinct between *S. verrucosum* and *S. phureja* (Gong *et al.* 2012; Zhang *et al.* 2014). To precisely determine the centromeres and compare these structures between the 2 species, we used publicly available ChIP-seq data generated against the CENH3 protein. The sequence reads from *S. verrucosum* (SRR18548893) and *S. phureja* (SRR18548894) were aligned to their genomes. Strong signals were observed on each chromosome, and the mapping rates were high (>97%) and comparable between the genomes of the 2 species, indicating that the centromere sequences were properly assembled in both genomes (Supplementary Table 1, Supplementary Fig. 3 and Fig. 2a). It was noted that 17.2% of the mapped reads were aligned to unanchored contigs, suggesting that some centromere sequences could not be assembled into chromosomes, possibly due to their highly repetitive nature.

In accordance with a previous report (Zhang *et al.* 2014), centromere regions showed little conservation between the sequences of *S. verrucosum* and *S. phureja* (Supplementary Fig. 4). In particular, the centromere position on chromosome 12 differed between *S. verrucosum* and *S. phureja* indicating that a massive rearrangement occurred during their speciation (Fig. 2b).

### Gene prediction

**Fig. 3.**
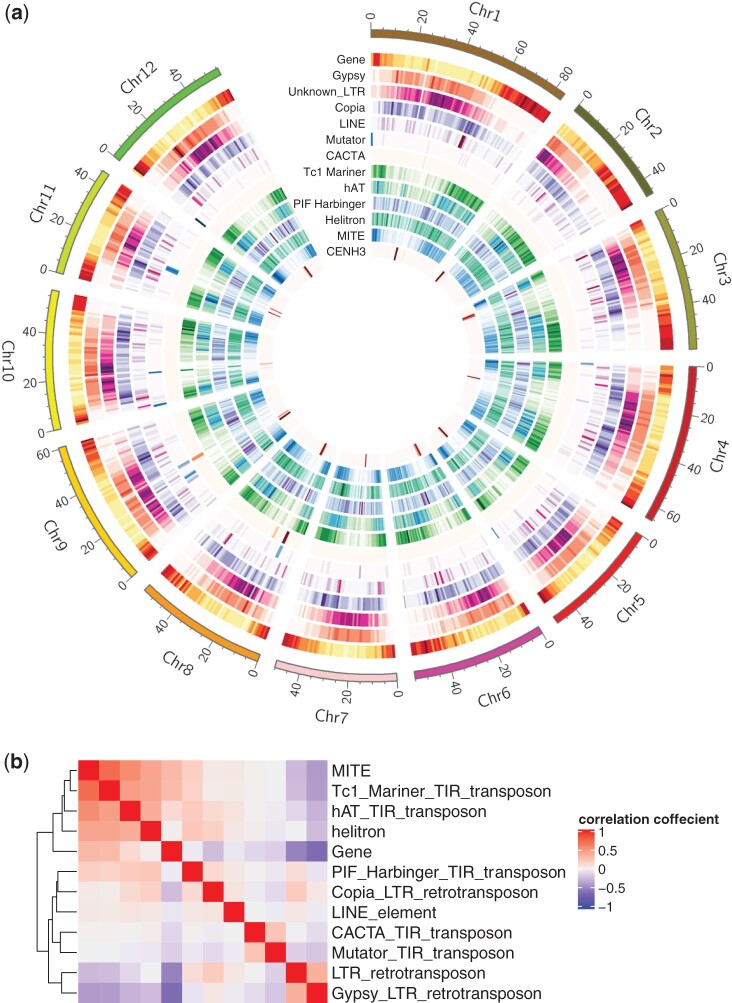
Chromosomal distribution of genes and transposons (a) and the correlations of their locations (b).

Since TEs are one of the major forces driving genome evolution (Hosaka and Kakutani 2018), the quantity and diversity of TEs were analyzed using the EDTA transposon annotation pipeline. TEs accounted for approximately 61.8% (482.1 Mb) of the *S. verrucosum* genome, among which LTR-type retrotransposons accounted for 39.9%, and terminal inverted repeat (TIR)-type transposons accounted for 9.7% (Supplementary Table 2). MITEs, which are nonautonomous derivatives of TIR-type transposons, were identified in 20.5% of the TIR-type transposons. The most abundant TEs were Gypsy elements (24.2%), as reported previously in other *Solanum* species (Aversano *et al.* 2015; Gaiero *et al.* 2019; Hosmani *et al.* 2019). Putative protein-coding genes were searched in the genome using the MAKER pipeline, and their functions were predicted using the Hayai-Annotation Plants v2 pipeline. As a result, 64,294 genes were predicted, and 46,904 genes were functionally annotated. Their chromosomal distributions and the correlations of their locations are shown in Fig. 3. The genes were densely distributed in telomeric and subtelomeric regions. Some class II transposons, such as Tc1_Mariner, hAT, helitron, and MITEs, were distributed in a pattern similar to that of genes. In contrast, Gypsy and unknown LTR retrotransposons were densely distributed in pericentromeric and centromeric regions. Similar distribution patterns have been reported in *S. phureja* DM v4.03 (Zavallo *et al.* 2020).

### Assembly completeness and quality assessment

The LAI score used to measure assembly completeness was 11.97, which was slightly lower than that of DM v6.1 but much higher than that of DM v4.04 (LAI scores of 13.56 and 7.87, respectively; Pham *et al.* 2020). Higher LAI scores correspond to more complete genome assemblies, and genome LAI scores between 10 and 20 are considered to indicate reference genome quality (Ou *et al.* 2018). Thus, the *S. verrucosum* genome was highly contiguous and is categorized as showing reference genome quality.

The quality of the gene predictions was assessed using the Benchmarking Universal Single-Copy Orthologs (BUSCO) database. Among 5,950 BUSCOs that are conserved in Solanales species, the number of complete BUSCOs identified was 5,759 (96.8%) in genome mode and 5,490 (92.3%) in protein mode. The number of missing BUSCOs identified in protein mode was 272 (4.6%), which was almost equivalent to the numbers found in the genomes of the other 5 species, ranging from 2.6% in *S. phureja* to 15.4% in *S. tuberosum* (Supplementary Table 3). Thus, most of the known BUSCOs were identified in the *S. verrucosum* genome, demonstrating robust representation of protein-coding genes.

### Synteny and phylogenetic analyses

**Fig. 4.**
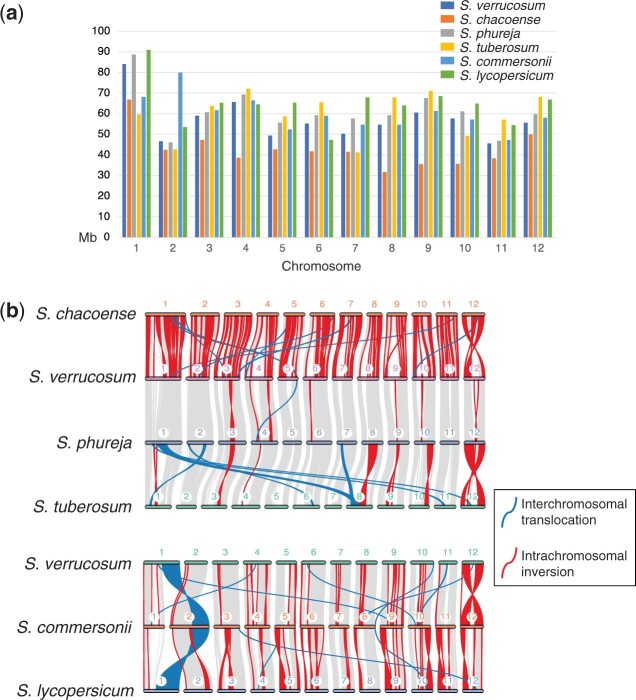
Size and structural differences among 6 species genomes. a) Chromosome size in Mb. b) Synteny plot between *S. verrucosum* and the other species.

Structural variation and gene similarity were compared between the *S. verrucosum* genome and the other 5 genomes. The chromosome lengths varied by species (Supplementary Table 4 and Fig. 4a). Each of the *S. chacoense* chromosomes except for the chromosome 7 was shorter than the corresponding chromosomes of the 5 other species, likely because *S. chacoense* showed the largest number of unanchored sequences (Leisner *et al.* 2018). The highest size similarity was observed between *S. verrucosum* and *S. phureja*, with a Pearson’s correlation coefficient of 0.978.

The gene collinearity analyses between *S. verrucosum* and the other 5 species showed putative inversions and interchromosomal translocations, indicating that genome rearrangements have occurred (Fig. 4b). The *S. verrucosum* genome was most syntenic to the *S. phureja* genome. Furthermore, all genomes except for that of *S. commersonii* showed similar gene synteny. The *S. commersonii* genome showed a large translocated segment on chromosome 2, which might indicate that a unique genome rearrangement occurred in this species, or this could be a result of simple misassembly.

**Fig. 5.**
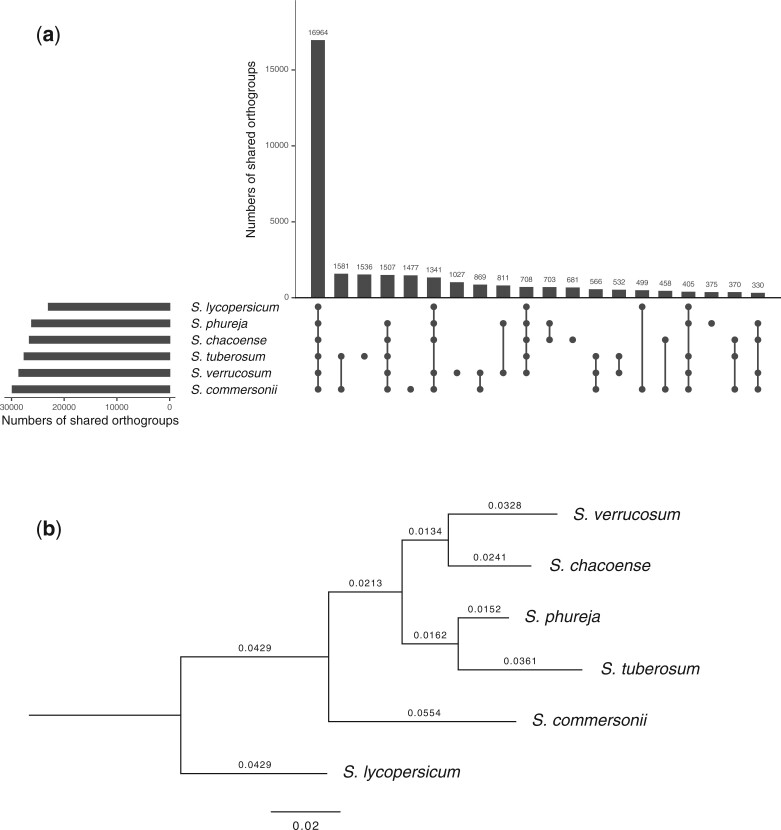
Orthologous relationships among 6 species genomes. a) UpSet plot of the shared orthogroups among 38,937 orthogroups assigned from a total of 288,020 genes identified in the 6 species genomes. The top 20 orthogroup intersections are shown. b) OrthoFinder-generated phylogenetic tree constructed using 16,964 orthogroups present in all 6 species. *S. lycopersicum* was used as the outgroup species.

The analysis of orthologous relationships using OrthoFinder showed that 288,020 genes (90.1%) among the 319,562 genes identified in the 6 species were assigned to 38,937 orthogroups (Supplementary Table 5). Among the 38,937 orthogroups, 16,964 (43.6%) were present in all the species analyzed, while only 1,027 (2.6%) were present in *S. verrucosum* (Fig. 5a). These *S. verrucosum*-specific orthogroups included 7,103 genes, of which 77.4% lacked functional annotations and 8.9% had similarities to the genes encoded in TEs based on the Hayai-Annotation Plants v2 pipeline. *S. verrucosum* presented the second largest number of shared orthogoups (28,568) after *S. commersonii*.

The species phylogeny was inferred from the similarity of the 16,964 orthogroups present in all the 6 species using OrthoFinder with the Species Tree inference from All Genes (STAG) algorithm (Emms and Kelly 2018). *S. commersonii* was distantly related among tuber-bearing species (Fig. 5b). In tuber-bearing *Solanum* species, the interspecific crossing barrier is explained by the Endosperm Balance Number (EBN) hypothesis (Johnston *et al.* 1980; Ehlenfeldt and Ortiz 1995). According to this hypothesis, a 2:1 ratio of maternal to paternal EBN in the endosperm is necessary for normal endosperm development (Johnston *et al.* 1980). *S. commersonii* shows an EBN of 1, whereas most of the other A-genome diploid species show an EBN of 2 (Ehlenfeldt and Hanneman 1988; Hanneman 1994). An imbalanced EBN causes endosperm abortion following interspecific hybridization, which is one of the major reproductive barriers among potato species (Johnston *et al.* 1980; Hawkes and Jackson 1992; Hanneman 1999). *S. verrucosum* was observed to be most closely related to *S. chacoense*, indicating that the 2 species have relatively similar gene sequences while frequent rearrangements by intrachromosomal inversions were observed (Fig. 4b). Interestingly, geographical distributions of the 2 species are most distant among the A-genome species (Hawkes 1990).

### Conclusions

We constructed a high-quality de novo assembly of the geographically isolated A-genome species *S. verrucosum* with a scaffold N50 of 55.2 Mb. The evaluation of variability within the A genome, including that of *S. verrucosum*, encompassed the geographic range of these species from the north (*S. verrucosum* in Mexico) to the south (*S. chacoense* in Argentina), which will be useful for understanding genomic differentiation among A-genome species. Since *S. verrucosum* has been considered a maternal progenitor of Mexican polyploid species (Hosaka *et al.* 1984; Spooner *et al.* 1991; Rodríguez and Spooner 2009; Sanetomo and Hosaka 2013), this whole-genome sequence will be a valuable resource for understanding polyploid evolution. Furthermore, the whole-genome sequence of *S. verrucosum* will facilitate the exploration of its unique crossing behaviors, such as self-compatibility and unilateral cross-compatibility (Hawkes 1990; Eijlander *et al.* 2000), which will help us understand its function as a bridging species (Hermsen and Ramanna 1976; Hamernik *et al.* 2001; Dinu *et al.* 2005; Jansky and Hamernik 2009; Bamberg *et al.* 2021) and will promote the introgression of useful traits from reproductively isolated Mexican diploid species into cultivated potatoes.

## Data availability

The raw DNA sequencing reads, genome assembly, and annotation have been deposited into the National Center for Biotechnology Information under BioProject Number PRJNA820895.

Supplemental material is available at *G3* online.

## Supplementary Material

jkac166_Supplementary_Figure_1

jkac166_Supplementary_Figure_2

jkac166_Supplementary_Figure_3

jkac166_Supplementary_Figure_4

jkac166_Supplementary_Tables

jkac166_Supplemetary_File_1

## Notes

The Hawkes (1990) classification system is tentatively adopted throughout the text.
