# Supplementary material for: A de novo genome assembly of Solanum verrucosum Schlechtendal, a Mexican diploid species geographically isolated from other diploid A-genome species of potato relatives
Source: G3 (Bethesda). 2022 Jul 1;12(8):jkac166. doi: 10.1093/g3journal/jkac166 (PMC9339273; doi:10.1093/g3journal/jkac166)
Supplement: jkac166_Supplementary_Figure_2 [file jkac166_supplementary_figure_2.pdf]

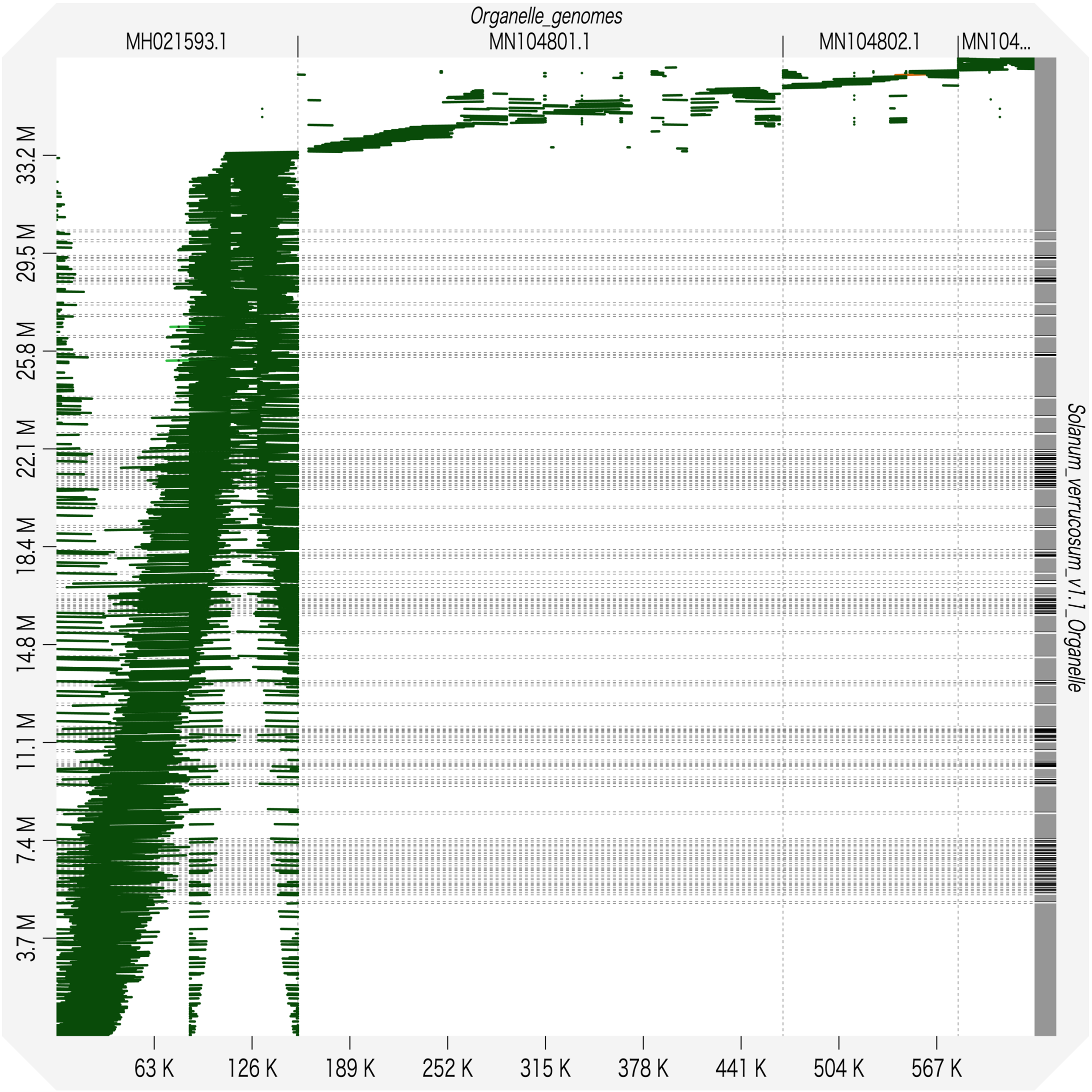

Supplementary Figure 2 Dot plot of *S. verrucosum* chloroplast genome (MH021593.1; Huang *et al*. 2019) and *S. tuberosum* cv. Désirée (MN104801, MN104802, and MN104803; Varré *et al*. 2019) mitochondrial genome against *S. verrucosum* contigs.
