# Supplementary material for: A de novo genome assembly of Solanum verrucosum Schlechtendal, a Mexican diploid species geographically isolated from other diploid A-genome species of potato relatives
Source: G3 (Bethesda). 2022 Jul 1;12(8):jkac166. doi: 10.1093/g3journal/jkac166 (PMC9339273; doi:10.1093/g3journal/jkac166)
Supplement: jkac166_Supplementary_Figure_3 [file jkac166_supplementary_figure_3.pdf]

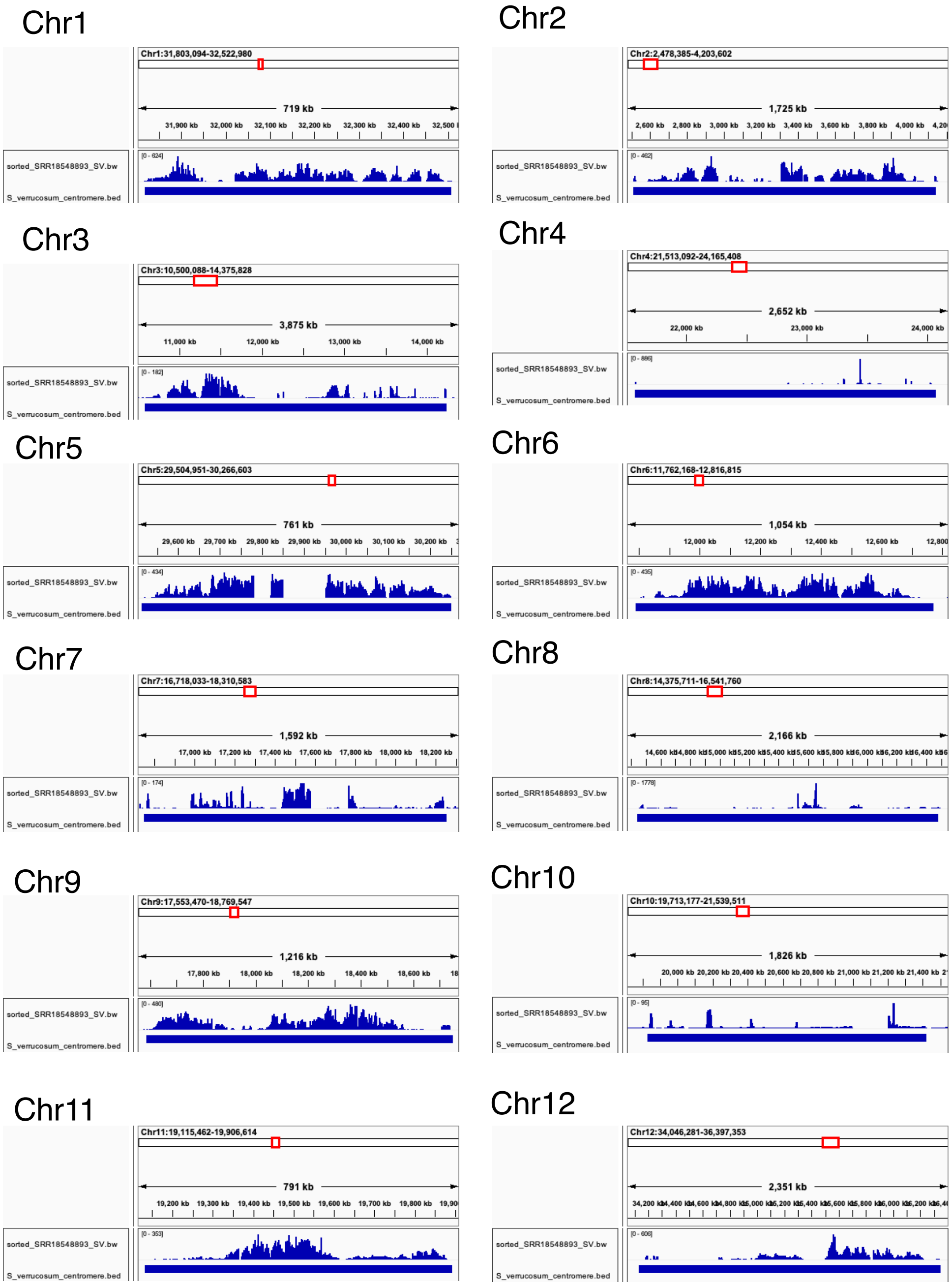

Supplementary Figure 3 ChIP-seq alignments in *S. verrucosum*. Bar chars indicate coverages of ChIP-seq against CENH3 protein. Blue boxes shown below are the defined centromere regions.
