# Supplementary material for: A de novo genome assembly of Solanum verrucosum Schlechtendal, a Mexican diploid species geographically isolated from other diploid A-genome species of potato relatives
Source: G3 (Bethesda). 2022 Jul 1;12(8):jkac166. doi: 10.1093/g3journal/jkac166 (PMC9339273; doi:10.1093/g3journal/jkac166)
Supplement: jkac166_Supplementary_Figure_4 [file jkac166_supplementary_figure_4.pdf]

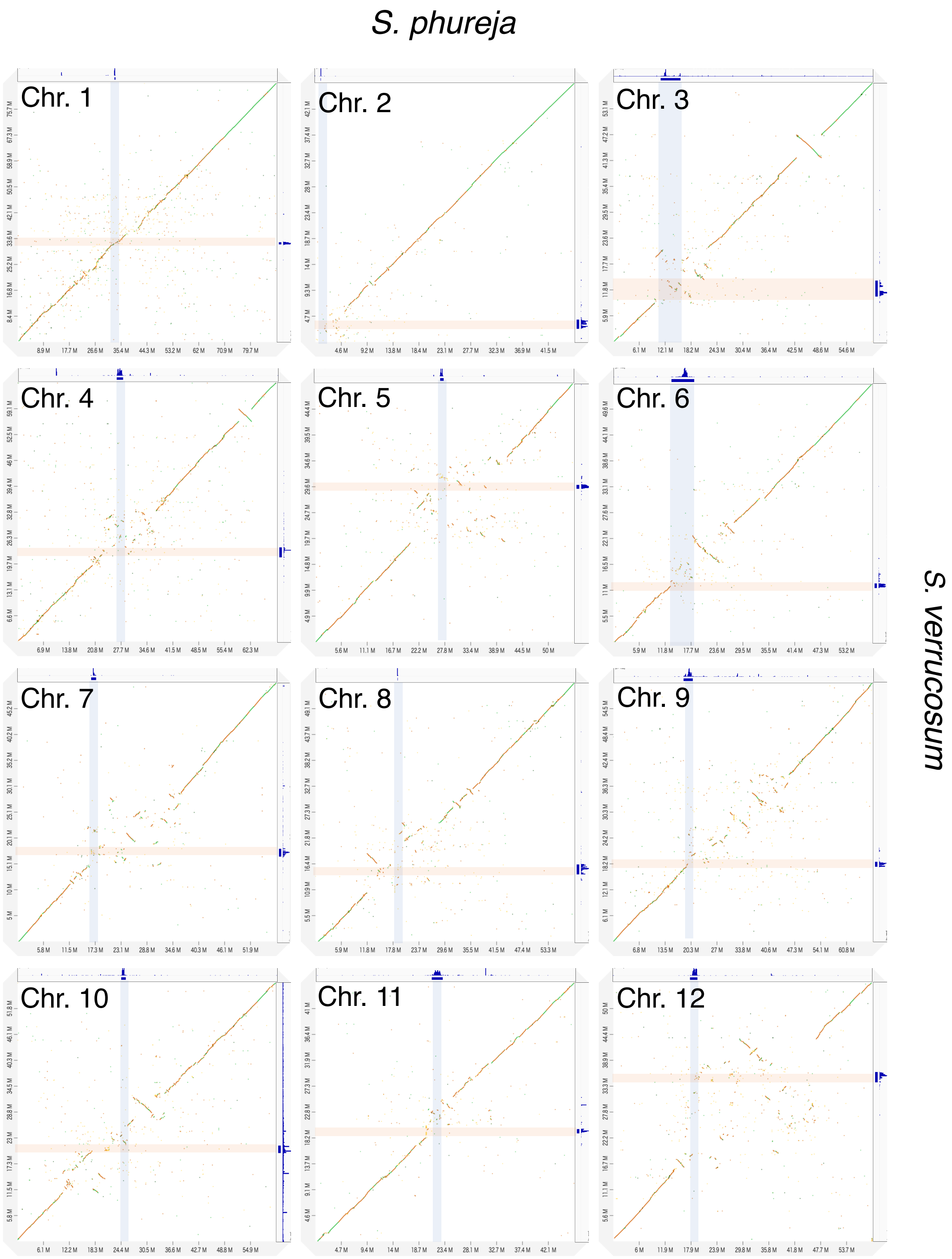

Supplementary Figure 4 Dot plots between *S. verrucosum* and *S. phureja*. ChIP-seq signals against CENH3 protein of *S. verrucosum* and *S. phureja* were shown at right and top of the plot, and highlighted in blue and orange, respectively.
