# Supplementary material for: A de novo genome assembly of Solanum verrucosum Schlechtendal, a Mexican diploid species geographically isolated from other diploid A-genome species of potato relatives
Source: G3 (Bethesda). 2022 Jul 1;12(8):jkac166. doi: 10.1093/g3journal/jkac166 (PMC9339273; doi:10.1093/g3journal/jkac166)
Supplement: jkac166_Supplementary_Tables [file jkac166_supplementary_tables.docx]

**Supplementary Table 1.** Statistics of CENH3 ChIP-seq

| Seq ID | Organism | Read length | Total Reads | Reads mapped to unique locations | Reads mapped to multiple locations | Mapping rate | Reads mapped to unanchored contigs | Mapping rate to unanchored contigs |
| --- | --- | --- | --- | --- | --- | --- | --- | --- |
| SRR18548893 | *S. verrucosum* | 50 bp (single-end) | 31,230,755 | 9,811,250 | 20,523,911 | 97.13% | 5,231,138 | 17.24% |
| SRR18548894 | *S. phureja* | 36 bp (single-end) | 43,082,181 | 12,802,801 | 29,499,266 | 98.19% | 4,305,290 | 10.18% |

**Supplementary Table 2.** Transposable element (TE) density

| Type | TE | Number^1)^ | Total size (bp) | Ratio (%) |
| --- | --- | --- | --- | --- |
| LTR | Copia | 57377 | 41002655 | 5.26% |
|  | Gypsy | 170963 | 188641838 | 24.19% |
|  | unknown | 124402 | 81282860 | 10.42% |
| TIR | CACTA | 44519 (1395) | 13990869 | 1.79% |
|  | Mutator | 80664 (13420) | 33375940 | 4.28% |
|  | PIF Harbinger | 13443 (1943) | 3824627 | 0.49% |
|  | Tc1_Mariner | 46935 (15075) | 10607108 | 1.36% |
|  | hAT | 42132 (14897) | 13858741 | 1.78% |
| non-LTR | LINE element | 2562 | 1086043 | 0.14% |
| non-LTR | helitron | 123269 | 40483249 | 5.19% |
| Repeat region |  | 138722 | 53973133 | 6.92% |
| Total |  | 844988 | 482127063 | 61.82% |

^1)^The number of miniature inverted-repeat transposable elements (MITEs) is shown in parentheses

**Supplementary Table 3.** BUSCO statistics

|  | *S. verrucosum* | *S. verrucosum* | *S. chacoense* | *S. phureja* | *S. tuberosum* | *S. commersonii* | *S. lycopersicum* |
| --- | --- | --- | --- | --- | --- | --- | --- |
| Mode | Genome | Protein | Protein | Protein | Protein | Protein | Protein |
| Complete BUSCOs | 5759 (96.8%) | 5490 (92.3%) | 5655 (95.0%) | 5679 (95.5%) | 4868 (81.8%) | 5371 (90.3%) | 5511 (92.6%) |
| Complete and single-copy BUSCOs | 5642 (94.8%) | 5319 (89.4%) | 4053 (68.1%) | 4090 (68.7%) | 4644 (78.1%) | 5145 (86.5%) | 5391 (90.6%) |
| Complete and duplicated BUSCOs | 117 (2.0%) | 171 (2.9%) | 1602 (26.9%) | 1589 (26.7%) | 224 (3.8%) | 226 (3.8%) | 120 (2.0%) |
| Fragmented BUSCOs | 33 (0.6%) | 188 (3.2%) | 103 (1.7%) | 116 (2.0%) | 164 (2.8%) | 190 (3.2%) | 169 (2.8%) |
| Missing BUSCOs | 158 (2.6%) | 272 (4.6%) | 192 (3.2%) | 155 (2.6%) | 918 (15.4%) | 389 (6.5%) | 270 (4.5%) |

**Supplementary Table 4.** Comparison of total assembly size (Mb) among related genomes

|  | *S. verrucosum* | *S. chacoense* | *S. phureja* | *S. tuberosum* | *S. commersonii* | *S. lycopersicum* |
| --- | --- | --- | --- | --- | --- | --- |
| Chromosome 1 | 84.1 | 66.8 | 88.6 | 59.6 | 68.1 | 90.9 |
| Chromosome 2 | 46.7 | 42.4 | 46.1 | 42.7 | 79.8 | 53.5 |
| Chromosome 3 | 59.0 | 47.1 | 60.7 | 63.7 | 61.6 | 65.3 |
| Chromosome 4 | 65.7 | 38.7 | 69.2 | 72.0 | 66.4 | 64.5 |
| Chromosome 5 | 49.4 | 42.7 | 55.6 | 58.5 | 52.4 | 65.3 |
| Chromosome 6 | 55.2 | 41.7 | 59.1 | 65.5 | 58.9 | 47.3 |
| Chromosome 7 | 50.2 | 41.5 | 57.6 | 41.1 | 54.6 | 67.9 |
| Chromosome 8 | 54.6 | 31.6 | 59.2 | 67.9 | 54.7 | 64.0 |
| Chromosome 9 | 60.5 | 35.4 | 67.6 | 71.0 | 61.2 | 68.5 |
| Chromosome 10 | 57.6 | 35.7 | 61.0 | 49.2 | 57.0 | 64.8 |
| Chromosome 11 | 45.5 | 38.3 | 46.8 | 57.0 | 47.2 | 54.4 |
| Chromosome 12 | 55.6 | 49.9 | 59.7 | 68.0 | 58.1 | 66.7 |
| Unanchored | 96.3 | 355.2 | 10.3 | - | 11.6 | 9.6 |
| Total size | 780.2 | 867.1 | 741.6 | 716.2 | 731.6 | 782.5 |

**Supplementary Table 5.** OrthoFinder statistics

| Statistics |  |
| --- | --- |
| Number of species | 6 |
| Number of genes | 319562 |
| Number of genes in orthogroups | 288020 |
| Number of unassigned genes | 31542 |
| Percentage of genes in orthogroups | 90.1 |
| Percentage of unassigned genes | 9.9 |
| Number of orthogroups | 38937 |
| Number of species-specific orthogroups | 5393 |
| Number of genes in species-specific orthogroups | 27799 |
| Percentage of genes in species-specific orthogroups | 8.7 |
| Mean orthogroup size | 7.4 |
| Median orthogroup size | 6 |
| G50 (assigned genes) | 8 |
| G50 (all genes) | 7 |
| O50 (assigned genes) | 8850 |
| O50 (all genes) | 10897 |
| Number of orthogroups with all species present | 16964 |
| Number of single-copy orthogroups | 7686 |
